# Acute hepatitis B virus infection with delayed appearance of hepatitis B core antibody in an immunocompromised patient: a case report

**DOI:** 10.1186/s13256-017-1264-9

**Published:** 2017-04-17

**Authors:** Nicholas Brousseau, Donald G. Murphy, Vladimir Gilca, Jacynthe Larouche, Sema Mandal, Richard S. Tedder

**Affiliations:** 1 CIUSSS de la Mauricie-et-Centre-du-Québec, 858 terrasse Turcotte, Trois-Rivières, Québec, G9A 5C5 Canada; 2 Institut national de santé publique du Québec, 945 av Wolfe, Québec, G1V 5B3 Canada; 3 Public Health England, 61 Colindale Ave, London, NW9 5EQ UK

**Keywords:** Hepatitis B virus infection, Immunosuppression, Rituximab, Anti-HBc, HBsAg

## Abstract

**Background:**

Despite the introduction of universal hepatitis B immunization programs worldwide, outbreaks of acute infection still occur in unimmunized individuals. A timely diagnosis of hepatitis B is necessary to ensure adequate clinical care and public health interventions that will reduce transmission. Yet, interpretation of hepatitis B serological markers can be complex. We present a case of hepatitis B with atypical markers, including delayed appearance of hepatitis B core antibody.

**Case presentation:**

A 62-year-old white woman was identified as a sexual contact of a male individual with acute hepatitis B virus infection. She had a history of recurrent low-grade non-Hodgkin lymphoma and had recently received immunosuppressive therapy. At baseline she had a negative serology and received three double doses (40 μg) of Engerix-B vaccine (hepatitis B vaccine) with a 0-month, 1-month, and 6-month schedule. One month following the last dose, hepatitis B surface antigen was positive in the absence of hepatitis B core antibody. The only sign of infection was a slight elevation of alanine aminotransferase enzymes a few months after first sexual contacts with the male individual. Hepatitis B virus infection was later confirmed despite the absence of hepatitis B core antibody. The development of hepatitis B core antibody was finally noted more than 6 months after the first positive hepatitis B surface antigen and more than 12 months after elevation of alanine aminotransferase enzymes. Immunosuppression including rituximab treatment was the most likely explanation for this serological profile. On her last medical assessment, she had not developed HBeAg seroconversion despite lower hepatitis B virus deoxyribonucleic acid levels with tenofovir treatment.

**Conclusions:**

When confronted with positive hepatitis B surface antigen in the absence of hepatitis B core antibody, consideration should be given to the possibility of both acute and persistent infection particularly in the setting of immunosuppression so that appropriate clinical management and public health interventions can take place. Given the increasing use of biologicals such as anti-tumor necrosis factor therapies either alone or with other immunosuppressive agents, this phenomenon may be encountered more frequently.

## Background

Since the introduction of universal immunization programs against hepatitis B virus (HBV) worldwide, the incidence of hepatitis B (HB) has largely declined [1]. In some low endemicity jurisdictions such as Quebec (Canada) where HB prevention includes two decades of school-based immunization, acute cases in birth cohorts eligible for immunization are extremely rare [2]. Nonetheless, cases are diagnosed each year and outbreaks still occur in unimmunized individuals [3, 4]. A timely diagnosis of acute HB is necessary to ensure adequate clinical care and public health interventions that will reduce transmission. Yet, interpretation of HB serological markers can be complex. Here we present a case of acute HBV infection with atypical markers, including delayed appearance of HB core antibody (anti-HBc). Very few studies have described the continued absence of anti-HBc during the early phase of the infection course [5].

## Case presentation

In March 2012, the case of a white man with acute HBV infection was reported to local public health authorities following investigation of a HB outbreak of nine acute cases associated with sexual transmission [4]. Among contacts of this person was a 62-year-old white woman with a history of recurrent low-grade non-Hodgkin lymphoma, diagnosed in 2006, with progression of the disease in November 2011. She received rituximab, cyclophosphamide, doxorubicin, vincristine, and prednisone (R-CHOP) treatment from November 2011 to March 2012. She also had a history of cholecystectomy, hysterectomy, operation for melanoma, and hypothyroidism. Concomitant medications included conjugated estrogens, levothyroxine, venlafaxine, and esomeprazole. She had sexual contacts with the male individual for the first time in February 2012 during his infectious period. No other risk factors for HBV infection were reported (no family history of HB or liver disease, no intravenous drug use, no unprotected sex with multiple partners, no travel in regions of high endemicity, no blood transfusion, no organ transplant). She was screened for HB surface antigen (HBsAg), ﻿HB surface antibody (anti-HBs)﻿, and anti-HBc and was found to be completely seronegative for all markers. According to provincial guidelines [6], this contact received three double doses (40 μg) of Engerix-B vaccine (HB vaccine) with a 0-month, 1-month, and 6-month schedule (April, May, and October 2012). Although the first sexual contact occurred approximately 1 month before diagnosis of the male individual, she did not receive HB immunoglobulins (HBIg) because there was no sexual contact within 14 days preceding diagnosis. No unprotected sexual contact occurred with the male individual after his diagnosis in March 2012.

Further serology was done 1 month after the last vaccine given to the female contact who was our patient (November 2012). HBsAg was positive with two different immunoassays (ADVIA Centaur XP Immunoassay System, Siemens Medical Solutions Diagnostics, Tarrytown, NY, USA and GS HBsAg Confirmatory Assay 3.0, Bio-Rad Laboratories, Redmond, WA, USA), anti-HBs was negative (ADVIA Centaur), and anti-HBc was completely unreactive with two different immunoassays (ADVIA Centaur and ORTHO HBc ELISA Test System, Ortho Clinical Diagnostics, Raritan, NJ, USA). Identical results were obtained for these markers in December 2012 and January 2013 (Table 1). The diagnosis of HB was finally confirmed in February 2013 when an HBV deoxyribonucleic acid (DNA) concentration of 3.5×10^9^ international units (IU)/mL was detected (COBAS AmpliPrep/COBAS TaqMan HBV Test v2.0, Roche Molecular Systems, Branchburg, NJ, USA). The November 2012 blood sample was tested a posteriori and it was also positive with an HBV DNA concentration of 1.8×10^9^ IU/mL.

**Table 1:** Laboratory results for the patient

	HBsAg	Anti-HBc	Anti-HBs	Anti-HBc IgM	HBeAg	Anti-HBe	HBV DNA	ALT (IU/L)	AST (IU/L)
March 2012 (pre-immunization)	neg.	neg.	neg.					63 (H)	45 (H)
November 2012 (post-immunization)	pos.^a^	neg.^a^	neg.				1.8×10^9^ IU/mL (done a posteriori)	(N)	(N)
December 2012	pos.^a^	neg.^a^	neg.					56 (H)	(N)
January 2013	pos.	neg.	neg.					(N)	(N)
February 2013	pos.	neg.	neg.	neg.	pos.	neg.	3.5×10^9^ IU/mL	(N)	(N)
March 2013	pos.	neg.	neg.		pos.	neg.	2.6×10^9^ IU/mL	(N)	(N)
June 2013	pos.	pos.	neg.		pos.	neg.	2.3×10^9^ IU/mL	53 (H)	54 (H)
October 2013	pos.	pos.	neg.		pos.	neg.	1.8×10^9^ IU/mL	81 (H)	52 (H)
January 2014	pos.	pos.	pos. (52.7 IU/L)		pos.	neg.	5.8×10^8^ IU/mL	65 (H)	44 (H)
April 2014	pos.	pos.	pos. (133.5 IU/L)		pos.	neg.	4.9×10^8^ IU/mL	44 (H)	34 (H)
August 2014	pos.	pos.	pos. (247.0 IU/L)		pos.	neg.	2.9×10^8^ IU/mL	43 (H)	37 (H)
December 2014 (tenofovir started in September 2014)	pos.	pos.	pos. (107.4 IU/L)		pos.	neg.	1.6×10^5^ IU/mL	(N)	(N)
March 2015	pos.	pos.	pos. (24.3 IU/L)		pos.	neg.	5.1×10^3^ IU/mL	(N)	(N)
June 2015	pos.	pos.	pos. (27.2 IU/L)		pos.	neg.	7.2×10^2^ IU/mL	(N)	(N)
September 2015	pos.	pos.	pos. (13.8 IU/L)		pos.	neg.	7.1×10^2^ IU/mL	(N)	(N)

*Anti-HBe* Hepatitis B envelope antibody, *ALT* alanine aminotransferase, *anti-HBc* he﻿patitis B core an﻿tib﻿ody, *anti-HBs* hepatitis B surface antibo﻿dy, *AST* aspartate aminotransferase, *blank* no result available, *DNA* deoxyribonucleic acid, *H* elevated value, *HBeAg* Hepatitis B envelope antigen, *HBsAg* hepatitis B surface antigen, *HBV* hepatitis B virus, *IgM* Immunoglobulin M, *IU* international units, *L* Litre, *mL* Millilitre, *N* normal value, *neg.* negative, *pos.* positive

^a^Confirmed with a second assay

The complete viral genome sequence of this patient was obtained on an acute phase blood sample (December 2012) and a chronic phase blood sample (December 2014). The HBV genome sequences recovered from the acute and chronic phase sera were identical. The HBV genome was genotype A, subgenotype A2 (serotype adw2). It did not display HBsAg mutations associated with immunization breakthrough, nor mutations or deletions in the core region that could impact anti-HBc production or detection.

She did not present with symptoms of acute HB during the investigation period. The only possible sign of infection was a slight elevation of alanine aminotransferase (ALT) and aspartate aminotransferase (AST) enzymes from March to May 2012 (Table 2). The investigation to identify other potential causes for transaminase elevation was negative. A physical and neurological examination was normal without stigmata of liver disease. After confirmation of HBV infection she maintained high levels of HBV DNA and developed elevated transaminase concentrations in June 2013 when anti-HBc first appeared. Treatment with tenofovir 300 mg once daily was started in September 2014. Her HBV DNA levels decreased from 2.9×10^8^ IU/mL in August 2014 to 7.1×10^2^ IU/mL in September 2015. Laboratory tests such as ALT and AST were normal throughout the treatment period. On her last medical assessment, she was asymptomatic but had not developed HBeAg seroconversion. No progression of the lymphoma had been observed since November 2011 and no additional immunosuppressive treatment was offered.

**Table 2:** Alanine aminotransferase and aspartate aminotransferase test results for the patient

	ALT (IU/L)	AST (IU/L)
April 2008 to early March 2012 (26 samples)	N	N
March to May 2012 (2 samples)	H (45–63)^a^	H (45)
September 2012 to May 2013 (7 samples)	N^b^	N
June 2013 to August 2014 (7 samples)	H (35–81)	H (34–54)
October 2014 to September 2015^c^ (5 samples)	N	N

*ALT* alanine aminotransferase, *AST* aspartate aminotransferase, *H* elevated value, *IU* international units, *N* normal value

^a^Numbers inside the brackets represent lowest and highest values for ALT and AST during the time period

^b^One ALT test result was slightly elevated (56 IU/L) in December 2012 and other test results were normal

^c^Tenofovir started in September 2014

## Discussion

This report describes a case of documented acute asymptomatic HBV infection presenting with delayed appearance of anti-HBc. The development of anti-HBc was noted more than 6 months after the first positive HBsAg and according to aminotransferase concentrations anti-HBc might have appeared more than a year after infection. Recent immunosuppressive therapy including rituximab for a non-Hodgkin lymphoma was the most likely causal mechanism. Lower anti-HBc production has been reported in immunocompromised individuals [7]. Furthermore, during an active phase of replication, high levels of HB core antigen﻿﻿ (HBcAg) are released in the bloodstream and can induce HBcAg and anti-HBc immune complex formation. This atypical profile has been described during chronic infection in immunosuppressed individuals, including a report of 39 cases with a concomitant diagnosis of human immunodeficiency virus (HIV), transplantation, or systemic inflammatory disease with immunosuppressive treatment [8–11]. Yet, to the best of our knowledge, very few studies have described the continued absence of anti-HBc during the early phase of infection course and none in association with rituximab treatment [5]. This clinical presentation is of interest as an isolated HBsAg could be considered a false positive leading to non-recognition of the acute infection. Clinicians and public health professionals must be aware of the potential impact of immunosuppression on HB serological markers. This is particularly important in the context of increasing cancer prevalence with more people taking immunosuppressive therapies and broader use of anti-tumor necrosis factor (TNF) monoclonal antibodies such as rituximab for rheumatologic conditions [12]. When confronted with an isolated HBsAg, a careful analysis is required with extended serological evaluation. A timely recognition of the infection will improve clinical management of cases and allow adequate preventive measures among contacts.

In regards to differential diagnosis, an antigenemia to HB immunization was excluded as HBsAg persisted for more than 4 weeks after HB immunization [13]. Another possible explanation for this serological profile would be a defective HB mutant virus [14–16], but this hypothesis was rejected after analysis of the HB whole genome. Finally, a rare cause of isolated HBsAg is selective immune system defect leading to lack of anti-HBc production, but the development of anti-HBc approximately 1 year after chemotherapy discontinuation led to rejection of this hypothesis [17].

In regards to the three double doses of HB vaccine administered to our patient, the fact that they were given several weeks after exposure and to an immunocompromised individual could explain why they did not prevent infection. HBIg was not given because no sexual contact had occurred within 14 days preceding diagnosis of her partner. The post-exposure period during which HBIg is effective is not well known. According to the Centers for Disease Control and Prevention [1], it is unlikely that this period exceeds 14 days post-exposure, and HBIg are not recommended beyond this delay. Further studies are needed to determine the post-exposure period during which HBIg can protect against infection [18]. In the meantime, a careful analysis of HBIg relevance should be made for immunocompromised individuals as their response to HB vaccine is impaired and as they are at higher risk of chronic infection and complications [17, 19].

Some limitations should be mentioned. First, we could not determine precisely the moment at which the patient was infected. However, aminotransferase monitoring and clinical history strongly suggest that it occurred around February 2012. Second, the infection source could not be proven as the genotype of the index case was not available. Yet, it is highly probable that the two cases were linked given that no other risk factors were found for the patient and seroconversion was observed after sexual exposure.

## Conclusions

After HBV infection, long-term persistence of HBsAg antigenemia with delayed anti-HBc seroconversion may occur in immunosuppressed individuals. Given the increasing use of biologicals such as anti-TNF monoclonal antibodies either alone or with other immunosuppressive agents, this phenomenon may be encountered more frequently. Detection of HBsAg without anti-HBc does not necessarily indicate incubation and consideration should be given to the possibility of both acute and persistent infection, particularly in the setting of immunosuppression, so that appropriate clinical management and public health interventions can take place.

## Abbreviations

ALT: Alanine aminotransferase
Anti-HBc: Hepatitis B core antibody
Anti-HBs: Hepatitis B surface antibody
AST: Aspartate aminotransferase
HB: Hepatitis B
HBcAg: Hepatitis B core antigen
HBIg: Hepatitis B immunoglobulins
HBsAg: Hepatitis B surface antigen
HBV: Hepatitis B virus
IU: International units
TNF: Tumor necrosis factor
